# Natural paniceins from mediterranean sponge inhibit the multidrug resistance activity of Patched and increase chemotherapy efficiency on melanoma cells

**DOI:** 10.18632/oncotarget.4162

**Published:** 2015-06-01

**Authors:** Laura Fiorini, Marie-Aude Tribalat, Lucy Sauvard, Julie Cazareth, Enzo Lalli, Isabelle Broutin, Olivier P. Thomas, Isabelle Mus-Veteau

**Affiliations:** ^1^ Institut de Pharmacologie Moléculaire et Cellulaire, UMR 7275, Université Nice Sophia Antipolis, CNRS, Valbonne, France; ^2^ Institut de Chimie de Nice, UMR 7272, Université Nice Sophia Antipolis, CNRS, Faculté des Sciences, Nice, France; ^3^ Laboratoire de Cristallographie et RMN Biologiques, UMR 8015, CNRS - Faculte de Pharmacie, Paris, France; ^4^ Institut Méditerranéen de Biodiversité et d'Ecologie Marine et Continentale, UMR 7263, CNRS, IRD, Université Aix-Marseille, Université Avignon, Station Marine d'Endoume, Marseille, France

**Keywords:** Patched, chemotherapy resistance, natural sponge compounds, drug efflux antagonist, cancer

## Abstract

Multidrug resistance has appeared to mitigate the efficiency of anticancer drugs and the possibility of successful cancer chemotherapy. The Hedgehog receptor Patched is a multidrug transporter expressed in several cancers and as such it represents a new target to circumvent chemotherapy resistance. We report herein that paniceins and especially panicein A hydroquinone, natural meroterpenoids produced by the Mediterranean sponge *Haliclona (Soestella) mucosa*, inhibit the doxorubicin efflux activity of Patched and enhance the cytotoxicity of this chemotherapeutic agent on melanoma cells *in vitro*. These results are supported by the molecular docking performed on the structure of the bacterial drug efflux pump AcrB and on the Patched model built from AcrB structure. Docking calculations show that panicein A hydroquinone interacts with AcrB and Patched model close to the doxorubicin binding site. This compound thus appears as the first antagonist of the doxorubicin efflux activity of Patched. The use of inhibitors of Patched drug efflux activity in combination with classical chemotherapy could represent a novel approach to reduce tumor drug resistance, recurrence and metastasis.

## INTRODUCTION

The Hedgehog (Hh) signaling pathway regulates body patterning and organ development during embryo development. In adults, the Hh pathway is mainly quiescent with the exception of roles in tissue maintenance and repair, and its inappropriate reactivation has been linked to several human cancers. Activation of Hh signaling occurs when Hh binds to its receptor Patched, causing the translocation of GLI1/2A into the nucleus to activate target genes such as snail, bcl2 and cyclin D, leading to proliferation, EMT and cell survival [1]. Indeed, aberrant activation of the Hh signaling has been identified in many aggressive cancers (breast, lung, colorectal, ovarian, pancreatic cancers, melanoma or multiple myeloma [2, 3]), in particular in cells exhibiting resistance to chemotherapeutic agents such as cancer stem cells or tumor initiating cells. Recently, Yue and collaborators showed that the Hh signaling is critical for lung squamous cell carcinomas (SCC) recurrence, metastasis and resistance to chemotherapy, suggesting that inhibition of the Hh pathway is a potential therapeutic strategy for the treatment of lung SCC patients [4]. Several studies have shown that antagonizing the Hh signaling receptor Smoothened (Smo) could provide a way to interfere with tumorigenesis and tumor progression [2, 5–8]. Vismodegib, designed to selectively inhibit Hh signaling by targeting Smo, is a first-in-class investigational oral medicine for basal-cell carcinoma treatment [2, 9]. It has been reported that autocrine expression of Hh morphogens such as Sonic Hedgehog (Shh) is required for growth of some cancers [10, 11], and stromal cell-derived Shh can also activate the Hh pathway in tumors [12]. A Shh-specific monoclonal antibody (5E1) has been shown to inhibit the growth of several tumors, including small-cell lung carcinoma, by preventing Shh binding to its receptor Patched [13]. Moreover, the use of an antibody directed against one of the Patched extracellular domains involved in the interaction with Shh as well as peptides designed to compete with Shh for binding to Patched were shown to inhibit proliferation of pancreatic cancer cells [14, 15]. In addition to targeting tumors that have hyperactive Hh pathway themselves, antagonists of the Hh pathway could also affect growth of tumors that use Hh ligands to induce angiogenesis [16, 17] or recruit other types of stromal cells. Because adults can tolerate inhibition of the Hh pathway [18], specific inhibition of Hh signaling offers an efficient treatment for diverse cancers originating from aberrant Hh pathway activation.

Two different genes (*Patched 1* and *Patched 2*) encode homologues of the Drosophila Hh morphogen receptor Patched. Mice deficient in *Patched 2* are viable, but develop alopecia and epidermal hypoplasia and have increased tumor incidence in the presence of a mutant allele of *Patched 1*. Loss of *Patched 1*, in turn, results in complete activation of the Hh pathway, suggesting that *Patched 1* is the functional ortholog of Drosophila Patched [1]. The expression of Patched 1, here referred as Patched, is induced upon activation of the Hh pathway in several cancers: lung, breast, basal cells of the skin, melanoma, prostate, colon, brain [2, 19, 20] and myeloid leukemia [21, 22]. Moreover, recent studies propose Patched as an early marker of gastric and thyroid cancers [23, 24].

We recently discovered that the Hh receptor Patched has a drug efflux activity and contributes to the resistance of cancer cells to some chemotherapeutic agents [25]. Indeed, human Patched expressed in yeast conferred resistance to several chemotherapeutic agents used to treat metastatic cancers (doxorubicin, methotrexate, temozolomide, 5-FU) and increases doxorubicin efflux. This yeast model has been extended to fibroblasts (often used to study Hh signaling) and human cancer cell lines which endogenously express Patched such as melanoma cell lines. The presence of Shh, the ligand of Patched which induces Patched internalization and degradation, was shown to increase the accumulation of doxorubicin into these cells and its cytotoxicity. Altogether, these results suggest that the Hh receptor Patched participates to chemotherapy resistance, and they prompted us to propose Patched as a new target for anti-cancer therapy. Discovering compounds able to inhibit the drug efflux activity of Patched would then lead to an increase in the efficiency of chemotherapy and thus to a reduction of the risk of metastasis and recurrence for patients with cancers expressing Patched. We then generated innovative yeast- and cell-based screenings to identify molecules able to inhibit the drug efflux activity of Patched. For this purpose, we decided to screen natural compounds produced by marine sponges commonly found in the Mediterranean Sea. Indeed, sponge natural products have already been identified as promising and original leads for therapeutic applications [26–28], and the high biodiversity of marine sponges growing in the diverse Mediterranean ecosystems is a guarantee of a large chemodiversity, enabling us to explore a significant volume of a “bioactive” chemical space. In the present study, we show that four known paniceins isolated from the species *Haliclona (Soestella) mucosa* [29] significantly inhibit the resistance to the chemotherapeutic agent doxorubicin of yeast expressing human Patched. One of these compounds, namely panicein A hydroquinone (1), enhances the accumulation and the cytotoxicity of doxorubicin for two melanoma cell lines, and we show that these effects are due to the inhibition of Patched doxorubicin efflux activity.

## RESULTS

### Paniceins isolated from the sponge *Haliclona (Soestella) mucosa* are inhibitors of the resistance to doxorubicin of yeast expressing Patched

**Figure 1:**
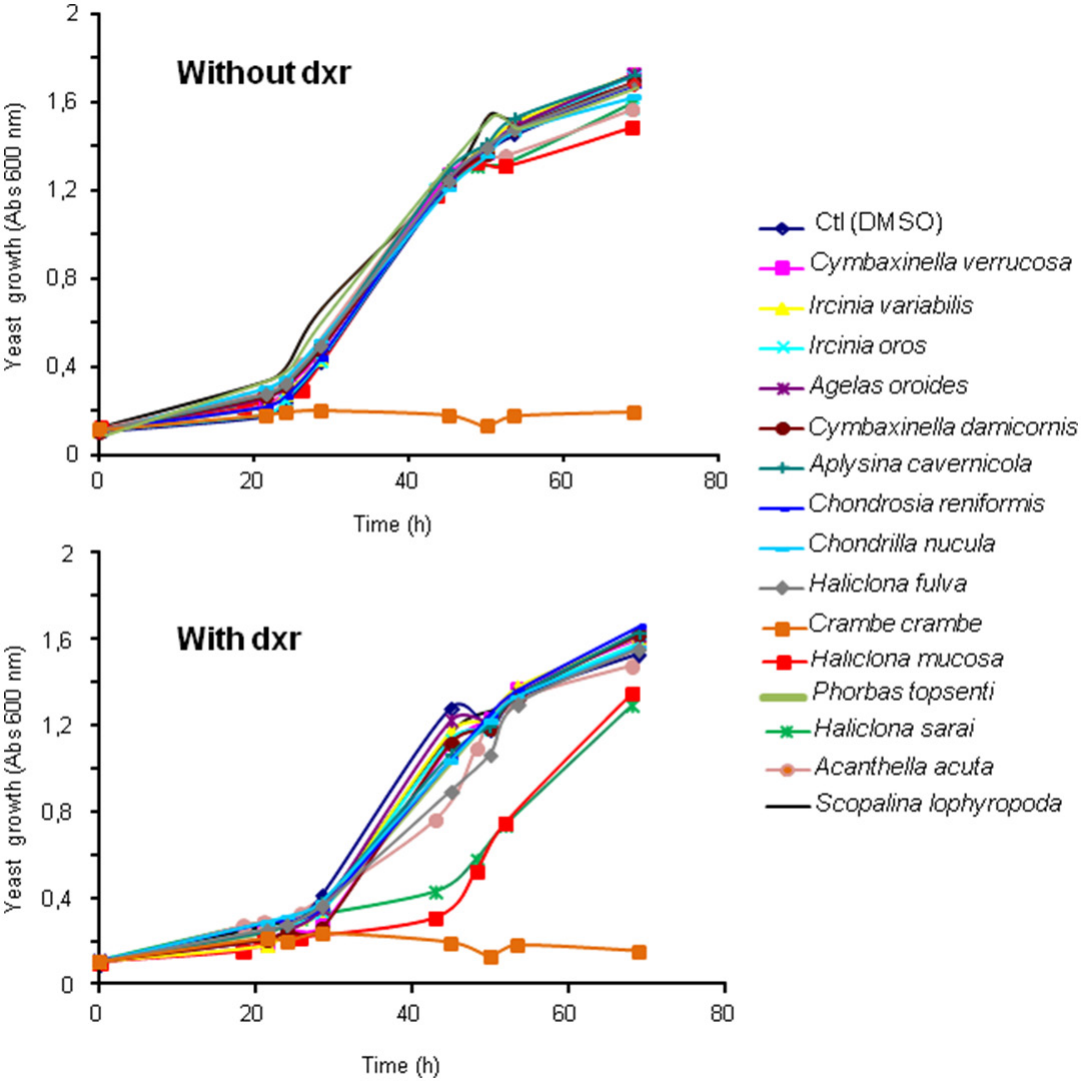
Effect of fifteen sponge fractions on the resistance of yeast expressing Patched to doxorubicin Yeast expressing Patched were grown in 96 well plates in the presence of 10 μg/mL of the methanolic fractions of fifteen Mediterranean sponges (*Cymbaxinella verrucosa, Cymbaxinella damicornis, Ircinia variabilis, Ircinia oros, Agelas oroides, Aplysina cavernicola, Haliclona (Soestella) mucosa, Phorbas topsenti, Chondrosia reniformis, Chondrilla nucula, Haliclona (Halichoclona) fulva, Crambe crambe, Haliclona (Rhizoniera) sarai, Acanthella acuta, Scopalina lophyropoda*) beforehand dissolved in DMSO, and in the presence or the absence of 10 μM of dxr. DMSO was used as control for yeast growth in the absence of the sponge fraction. The growth of yeast was measured by absorbance at 600 nm.

**Figure 2:**
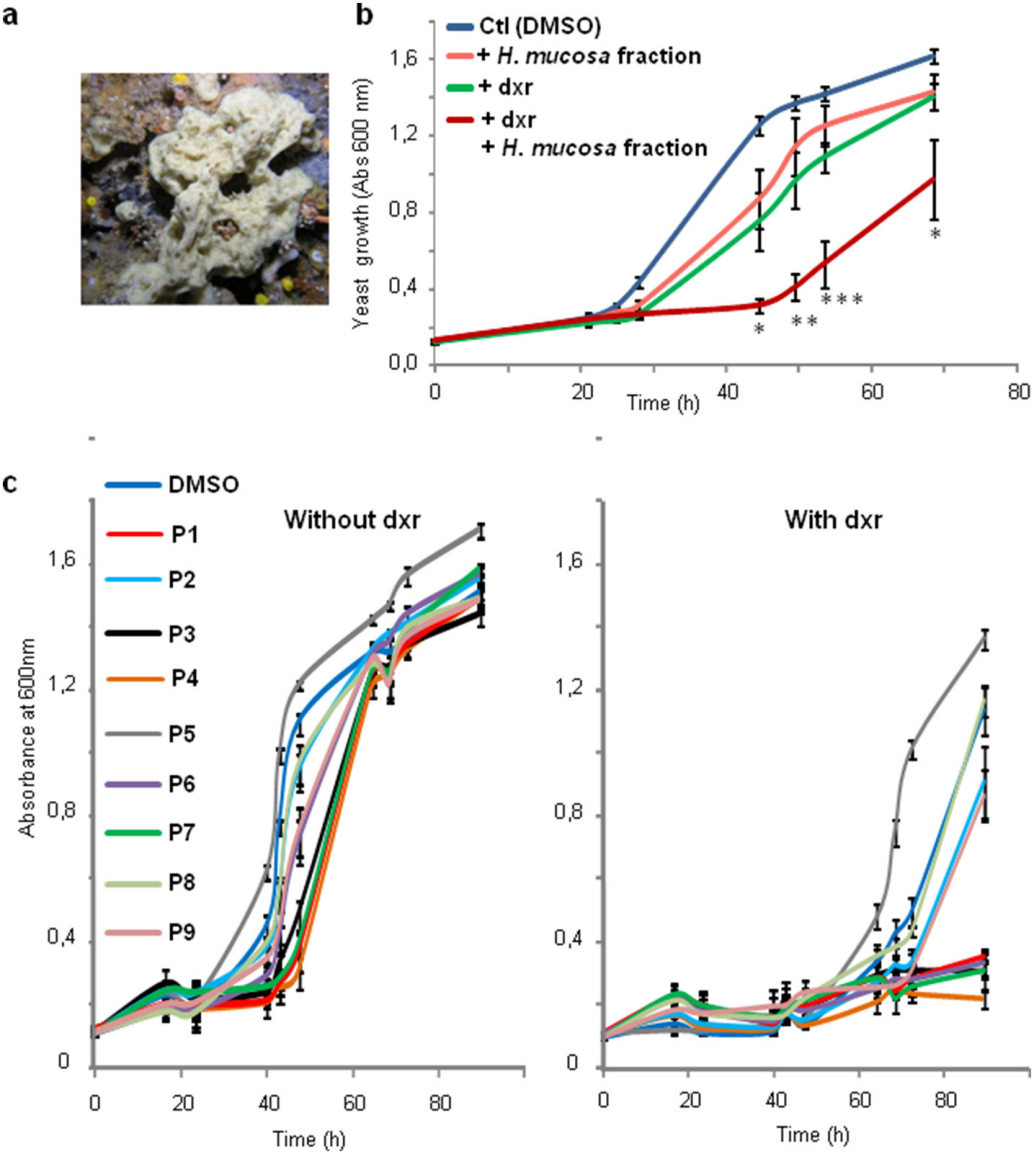
Haliclona mucosa extract inhibits resistance of yeast expressing Patched to doxorubicin **a.** Picture of the sponge *Haliclona mucosa*. **b.** Yeast expressing Patched were grown in 96 well plates in the presence of 10 μg/mL of the methanolic fraction of *Haliclona mucosa* crude extract dissolved in DMSO and in the presence or in the absence of 10 μM of dxr. DMSO at a dilution of 1/1000 corresponding to the amount of DMSO added with the sponge extracts was used in the control wells. The growth of yeast was measured by absorbance at 600 nm. The results shown are the mean +/− SEM of three independent experiments and were analyzed using the Student *t*-test in which significance is attained at *P* < 0.05 (*) (**: *P* < 0.005, ***: *P* < 0.0005) in comparison with the growth of yeast in the presence of dxr and DMSO. **c.** Compounds purified from *Haliclona mucosa* methanolic fraction were dissolved in DMSO at 10 mg/mL and added at a final concentration of 10 μg/mL to the growth medium containing or not 10 μM of dxr. DMSO at a dilution of 1/1000 corresponding to the amount of DMSO added with the purified sponge fractions was used in the control wells. The growth of yeast was measured by absorbance at 600 nm. The results shown are the mean +/− SEM of three independent experiments.

**Figure 3:**
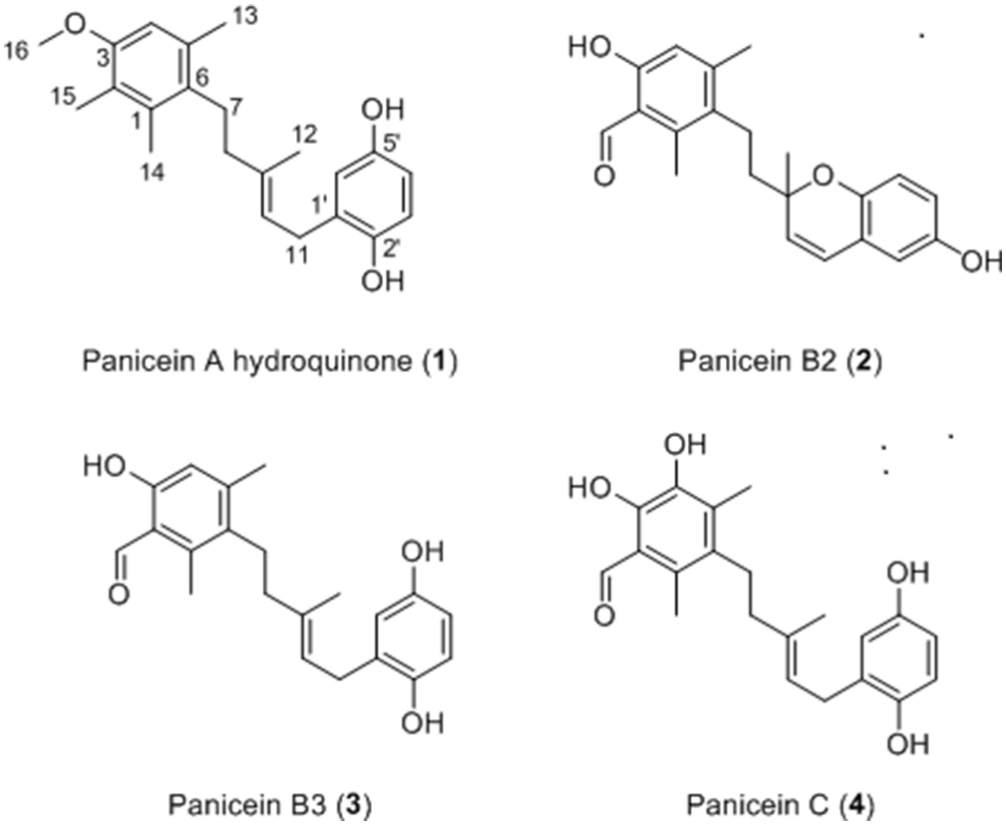
Structure of paniceins isolated and identified from *Haliclona (Soestella) mucosa* **1.** was identified as panicein A hydroquinone, **2.** was identified as panicein B2, **3.** was identified as panicein B3, **4.** was identified as panicein C.

**Figure 4:**
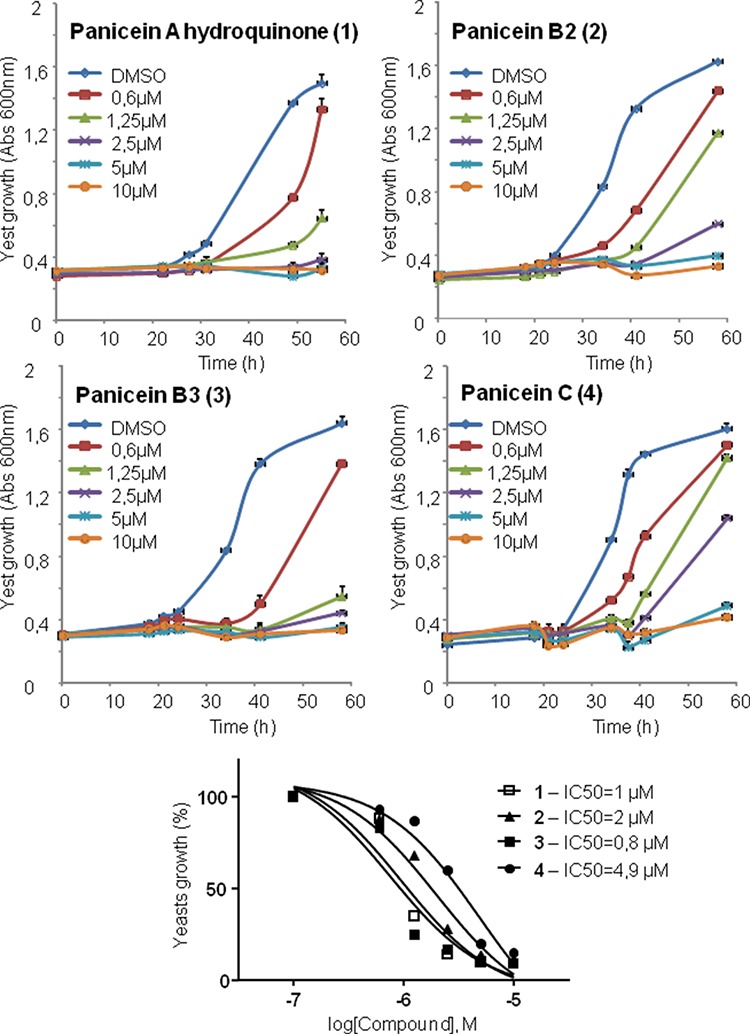
Panicein compounds purified from *Haliclona mucosa* inhibit resistance of yeast expressing Patched to doxorubicin Yeast expressing Patched were grown in 96 well plates. Panicein compounds purified from *Haliclona mucosa* methanolic fraction and dissolved in DMSO at 50 mg/mL were added at increasing concentrations to the growth medium containing 10 μM of dxr. DMSO at a dilution of 1/1000 was used in the control wells. The growth of yeasts was measured by absorbance at 600 nm. The results shown are the mean +/− SEM of four wells. IC_50_ were calculated using nonlin fit of log-dose vs response from Graph Pad Prism software.

In a previous study, we showed that the expression of human Patched allowed yeast to grow in the presence of a concentration of doxorubicin (dxr) that inhibits the growth of control yeast, indicating that Patched confers resistance to dxr [25]. From these results, we developed a screening test in 96-well plates to identify compounds capable of inhibiting the resistance of yeast expressing human Patched to dxr. First, the methanolic fractions of fifteen representative Northwestern Mediterranean sponges (*Cymbaxinella verrucosa* [30], *Cymbaxinella damicornis, Ircinia variabilis, Ircinia oros, Agelas oroides, Aplysina cavernicola, Haliclona (Soestella) mucosa, Phorbas topsenti, Chondrosia reniformis, Chondrilla nucula, Haliclona (Halichoclona) fulva, Crambe crambe, Haliclona (Rhizoniera) sarai, Acanthella acuta, Scopalina lophyropoda*) were obtained after C18 Solid Phase Extraction (SPE) of a MeOH/DCM 1:1 extract of each sponge. These fractions were subsequently dissolved in DMSO and added at a final concentration of 10 μg/mL to yeast expressing Patched in a medium supplemented or not with dxr (Figure 1). The methanolic fraction obtained from the sponge *Crambe crambe* completely repressed the growth of yeast expressing Patched even in the absence of dxr and appeared to be cytotoxic for yeast. The methanolic fractions obtained from *Haliclona (Soestella) mucosa* and *Haliclona (Rhizoniera) sarai* were the only that clearly inhibit the growth of yeast expressing Patched in the presence of dxr without significant effect in the absence of dxr. We decided to focus our study on the methanolic fraction obtained from *Haliclona (Soestella) mucosa* (Figure 2a) which significantly inhibited the resistance of yeast expressing Patched to dxr with only a small effect on basal yeast growth (in the absence of dxr) (Figure 2b). In order to identify the compounds responsible for this bioactivity, the methanolic fraction obtained from *Haliclona (Soestella) mucosa* was purified by C_18_ preparative HPLC to yield 9 peaks (P1-P9) (Supplementary Figure 1). Compounds present in these collected peaks were added singly to the yeast growth medium in the presence or in the absence of dxr. Five of these peaks (namely P1, P3, P4, P6 and P7) were shown to strongly inhibit the resistance to dxr of yeast expressing Patched (Figure 2c). The effects of P2 and P9 on yeast growth were lower, and P8 had no effect. Surprisingly, peak P5 increased yeast growth in the presence of dxr suggesting that this compound enhances yeast resistance to dxr. Comparison of the NMR data of these peaks with literature allowed the identification of four compounds P3, P4, P6 and P7 and confirmed their purity (>95%) (Supplementary Figure 2), while the other peaks remained unidentified because they were found as complex mixtures. The identified compounds are meroterpenoids members of the panicein family, namely panicein A hydroquinone (1, P7) [31], panicein B2 (2, P6), panicein B3 (3, P4), and panicein C (4, P3) [32] (Figure 3). Dose-responses of these compounds on the growth of yeast expressing Patched in the presence of dxr have been performed providing IC50 values of about 1 μM for 1, 2 μM for 2, 0.8 μM for 3 and 4.9 μM for 4 (Figure 4).

### Panicein A hydroquinone increases the cytotoxicity of doxorubicin on melanoma cells

An analysis extracted from Human Protein Atlas website (http://www.proteinatlas.org/ENSG00000185920-PTCH1/cancer) shows that the protein Patched is expressed in several cancers and in particular in melanoma, which exhibit a strong expression of Patched and no expression in healthy tissue (Supplementary Figure 3a). Data from the cancer microarray database and web-based data-mining platform ONCOMINE (https://www.oncomine.org/) [33] indicate that Patched mRNAs are expressed in biopsies from 83 melanoma samples over 154, both in primary site and in metastasis (Supplementary Figure 3b). Therefore, two melanoma cell lines were chosen to measure the effect of paniceins purified from *Haliclona (Soestella) mucosa* on dxr cytotoxicity. The MEWO cell line is derived from a melanoma metastatic site (lymph node tissue), and the A375 cell line is derived from a human malignant melanoma and carries the BRAFV600E mutation. These two cell lines express the protein Patched as shown by western-blotting and immunofluorescent labeling (Supplementary Figure 3c).

**Figure 5:**
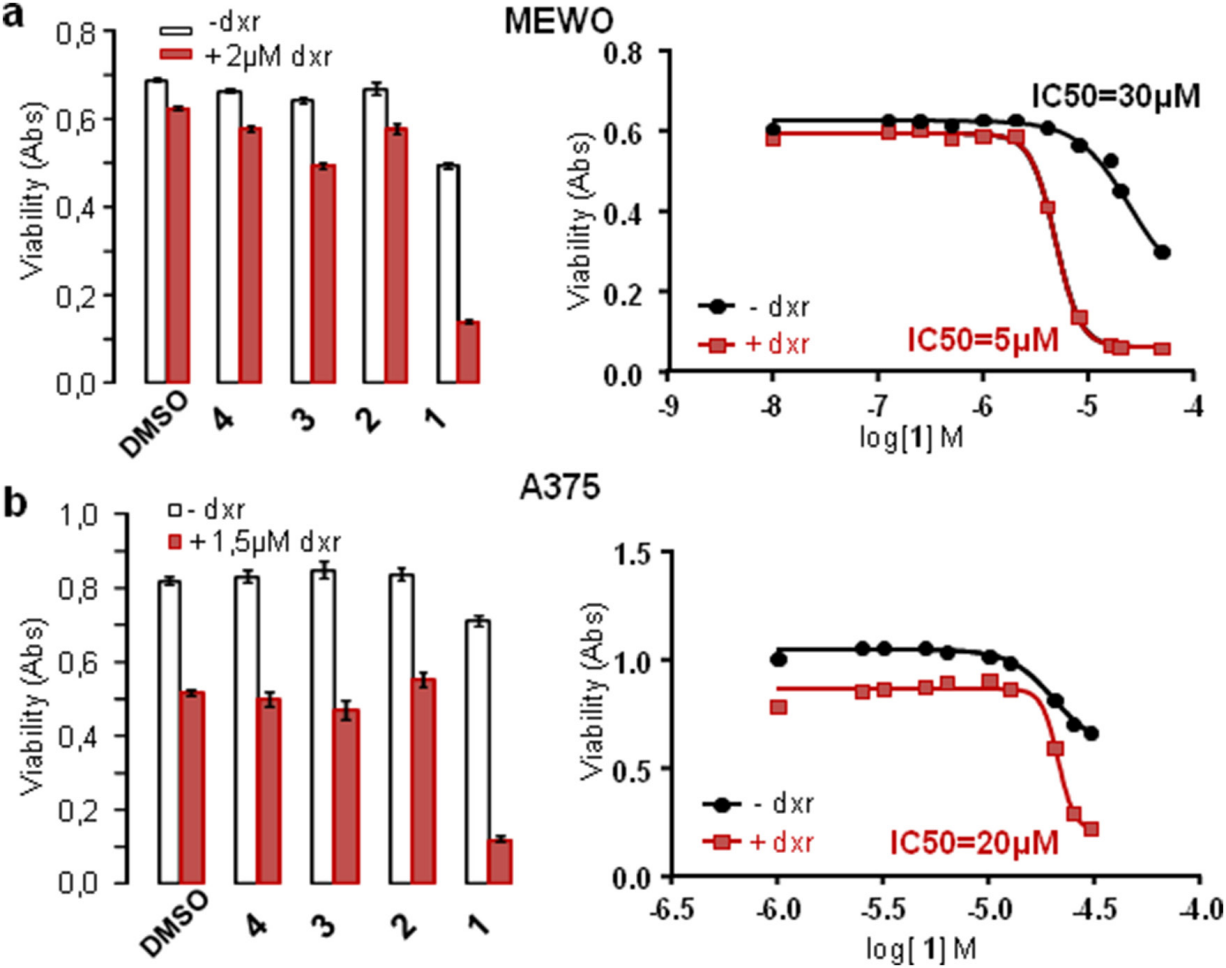
Panicein A hydroquinone increases doxorubicin cytotoxicity on melanoma cells Melanoma cell lines were treated with paniceins (10 μM MEWO and 25 μM for A375 (left histograms) or increasing concentrations for IC_50_ measurements (right part)) or DMSO (control), with (red) or without (white) dxr. Cell viability was measured after 24 h treatment. IC_50_ were calculated from the mean of at least three experiments for each cell line using nonlin fit of log-dose vs response from Graph Pad Prism software.

Cells were treated separately with the four paniceins 1–4, with or without dxr, during 24 hours before cell viability measurement (Figure 5). The average of experiments indicated that compound 1 strongly increased cell mortality induced by dxr on MEWO cells (5 to 8 times) and on A375 cells (2 to 3 times). Dose-responses of Panicein A hydroquinone (1) on cell viability have been performed providing IC50 values in the presence of dxr of about 5 μM and 20 μM on MEWO and A375 cells respectively, while in the absence of dxr, this molecule was weakly cytotoxic with IC50 superior to 30 μM.

**Figure 6:**
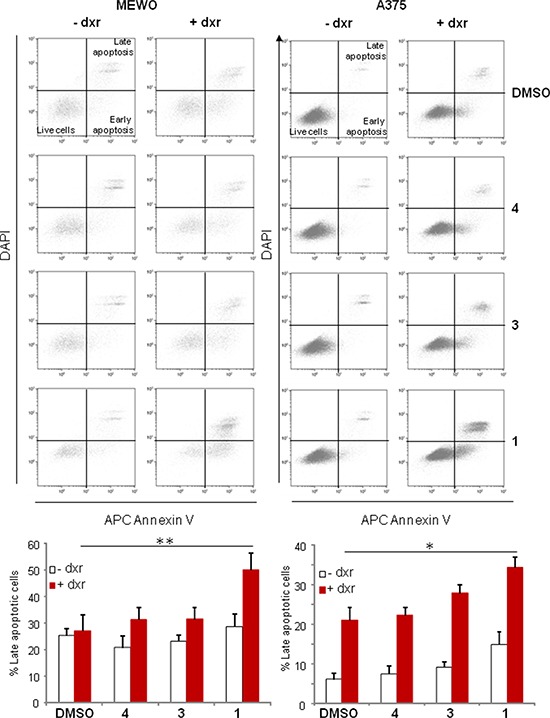
Panicein A hydroquinone strongly increases the number of apoptotic melanoma cells Cells were sampled after 24 h treatment with DMSO, paniceins and/or dxr, and apoptosis determined via AnnexinV and DAPI co-staining. Cells in early apoptosis are AnnexinV positive and DAPI negative, and cells in late apoptosis are AnnexinV and DAPI double positive. Histogram represents the mean percentage (+/− SEM) of cells in late apoptosis from three independent experiments and were analyzed using the Student *t*-test in which significance is attained at *P* < 0.05 (*) (**: *P* < 0.005).

Flow cytometry experiments carried out using Annexin V and DAPI labeling indicated that the addition of 1 to dxr treatment significantly increased the percentage of cells in apoptosis both for MEWO and A375 cells (Figure 6). These data are in good agreement with the dxr cytotoxicity increase effect of this compound.

### Panicein A hydroquinone inhibits doxorubicin efflux

**Figure 7:**
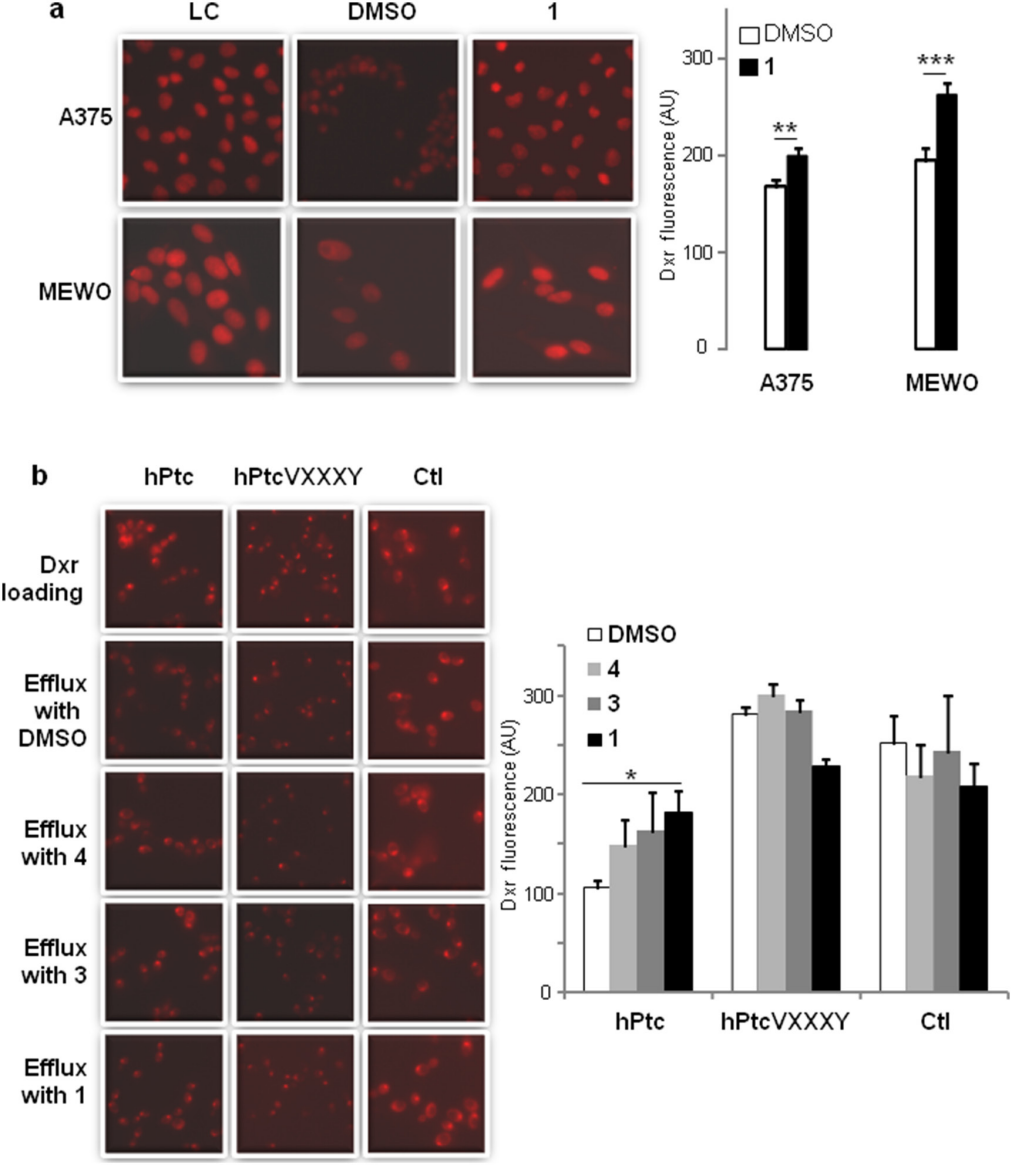
Paniceins inhibit doxorubicin efflux **a.** MEWO and A375 cells were incubated for 2 hours with dxr and immediately fixed for dxr loading control (LC), or incubated 30 min with buffer supplemented with DMSO or 10 μM of 1 and immediately fixed. Intracellular dxr fluorescence (red) was visualized by epi-fluorescence with 40X magnification. **b.** Yeast expressing wild-type Patched, mutant Patched G509VD513Y (VXXXY), and control yeast were incubated with dxr for 2 hours and immediately fixed for dxr loading control (LC), or resuspended in buffer supplemented with DMSO or paniceins 10 min and fixed. Samples were deposited on cover-slips and intracellular dxr fluorescence (red) was visualized by epi-fluorescence with 63X magnification. Histograms represent the intracellular dxr fluorescence quantification which was carried out using Image J software on more than 30 cells or yeasts from 3 different fields for each condition on 3 independent experiments. The results were analyzed using the Student *t*-test in which significance between dxr fluorescence in cells after efflux in the presence of DMSO (in white) or of 1 (in black) is attained at *P* < 0.05 (*) (**: *P* < 0.005, ***: *P* < 0.0005).

Western blot analysis performed on MEWO or A375 cells treated during 24 hours with compound 1 or with DMSO indicated that the treatment with panicein A hydroquinone had no effect on Patched expression or degradation in these cells (Supplementary Figure 4). Then, we hypothesized that the effect of 1 on the resistance and the cytotoxicity of dxr could be due to the inhibition of Patched dxr efflux activity. We took advantage of the natural fluorescence properties of dxr [34] to carry out dxr efflux measurements. Melanoma cells were loaded with dxr and fixed for loading control or incubated with efflux buffer containing DMSO (efflux control) or compound 1. Cells were fixed after 30 min and analyzed using cell imaging (Figure 7a). Fluorescence intensity quantification showed that dxr intracellular concentration was significantly higher (about 25%) when 1 was present in the efflux buffer for both A375 and MEWO cells. These results suggest that 1 inhibits dxr efflux from these melanoma cells.

In order to demonstrate that compound 1 inhibits dxr efflux by acting on Patched, we compared dxr efflux from yeast expressing Patched and control yeast. 2-deoxy-D-glucose was added in buffer during dxr loading and efflux in order to de-energize yeast and inhibit ATP-binding cassette (ABC) transporters which also contribute to dxr efflux in yeast. This enabled us to selectively study the dxr efflux activity of Patched. Dxr fluorescence measured in yeast expressing Patched after efflux in buffer containing DMSO was significantly lower than that measured in control yeast, consistently with the dxr efflux activity of Patched already described [25] (Figure 7b). Dxr concentration of yeast expressing Patched was significantly higher when 1 was present in the efflux buffer, unlike compounds 3 and 4 which exhibited no significant effect (Figure 7b). Results analysis indicated that 1 inhibited about 40% of the dxr efflux activity of Patched. None of these compounds have a significant effect on the dxr fluorescence of control yeasts. These results suggest that compound 1 is able to inhibit the dxr efflux activity of Patched. This was confirmed using yeast expressing a mutant of Patched: PatchedVXXXY. We previously described that the drug efflux activity of Patched was coupled to the proton motive force similarly to the bacterial efflux pumps from the RND family [35]. The GXXXD motif that corresponds to the proton transfer pathway in the RND bacterial drug efflux pumps is highly conserved in the fourth putative transmembrane segment of Patched [25, 36, 37]. We replaced glycine (G) in position 509 by a valine (V) and aspartic acid (D) in position 513 by a tyrosine (Y), and we observed that yeast expressing PatchedVXXXY are less resistant to growth inhibition by dxr than yeast expressing wild-type Patched [25]. According to these previous observations, yeast expressing PatchedVXXXY contained significantly more dxr after efflux than yeast expressing wild-type Patched (Figure 7b). The concentration of dxr in yeast expressing PatchedVXXXY was comparable to that in control yeast, confirming that the double mutation inhibited the dxr efflux activity of Patched. As expected, the presence of compounds 1, 3 or 4 in the efflux buffer had no effect on the dxr concentration of yeast expressing PatchedVXXXY in contrast to yeast expressing wild-type Patched (Figure 7b). These results support the hypothesis that panicein A hydroquinone (1) inhibits the dxr efflux activity of Patched.

### Panicein A hydroquinone presents a strong docking cluster close to the doxorubicin binding site of AcrB

**Figure 8:**
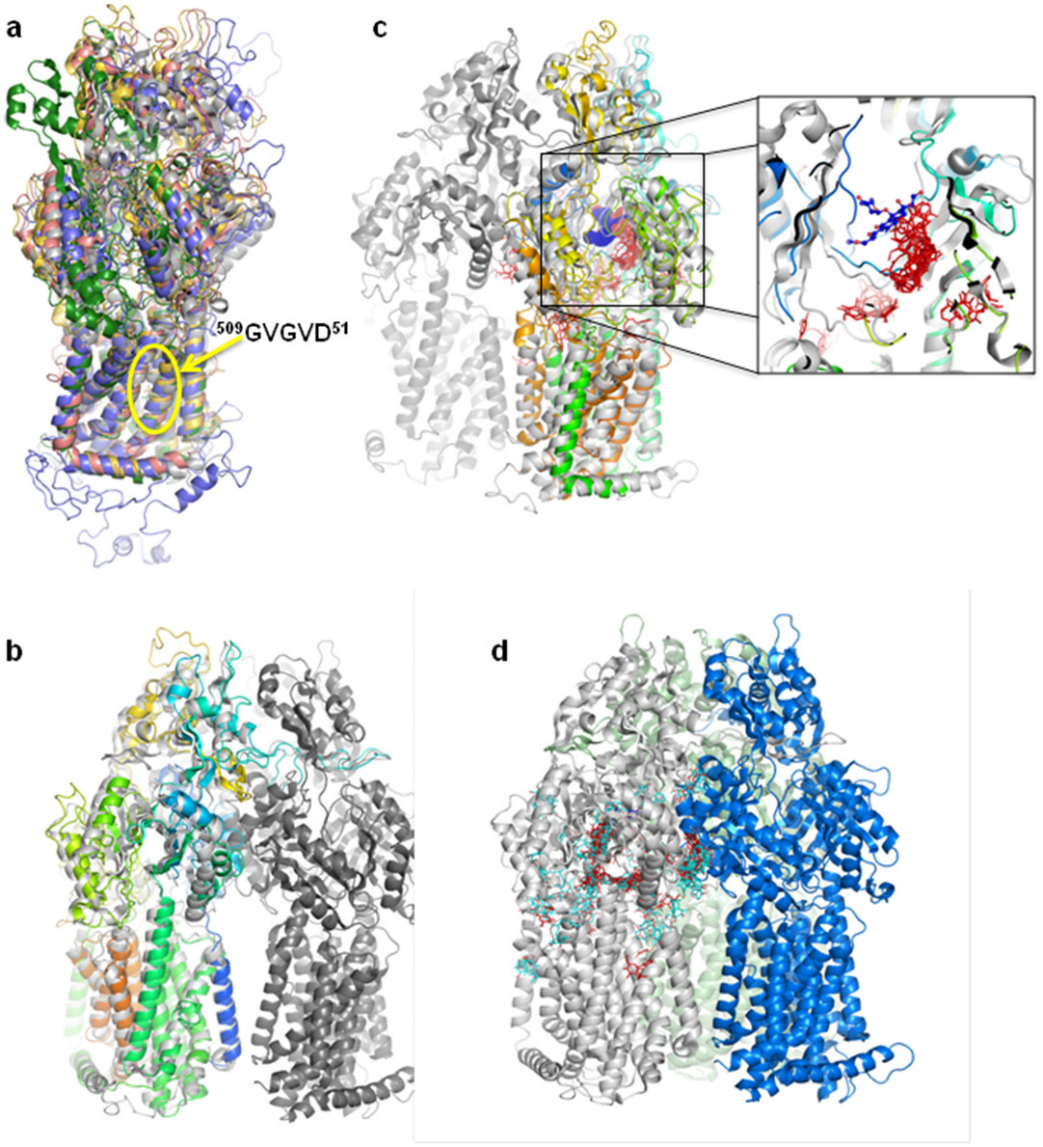
Panicein A hydroquinone presents a strong docking cluster close to the doxorubicin binding site in AcrB structure and in Patched structural model **a.** Superposition of the different models obtained for human Patched. The transmembrane domain is consistent between the different results, especially the residues GVGVD taking part of the proton transfer. **b.** Monomer of the final selected Patched model superposed on the AcrB structure. The trimer of AcrB is colored in three different greys, the Patched model is in rainbow color. **c.** Panicein A hydroquinone / AcrB docking results. All the panicein A hydroquinone possible positions are presented. The zoom corresponds to an enlargement of the dxr binding pocket. The proteins structures are colored as in (b), dxr is in blue, panicein A hydroquinone is in red. **d.** AcrB docking results of panicein C (light blue) and panicein A hydroquinone (red) presented on a monomer of the AcrB structure. The three monomers are colored in light grey, light green, and blue.

The different programs used for Patched model building converged to the models built with the drug efflux pumps from the RND family (AcrB, MexB, CusA) despite sequence identities between Patched and these proteins lower than 18% (36% of sequence similarity between human Patched and *E. coli* AcrB). The transmembrane region is the most conserved one. In particular, the sequence ^509^GVGVD^513^, corresponding to the proton transfer pathway, is perfectly aligned in all the models built (Figure 8a). The final Patched model results mainly from the structure of *Escherichia coli* AcrB as evidenced from the structural superposition (Figure 8b). The docking of two panicein molecules (1 and 4) was carried out on the structure of this RND transporter. Among the numerous AcrB structures present in the Protein Data Bank (PDB), the highest resolution one obtained without darpin nor substrate (PDB code 2GIF; [38]) was chosen as template and the results were compared with the AcrB structure in complex with dxr (PDB code: 2DR6; [39]). Those structures consist of three protomers, each of which has a different conformation corresponding to one of the three functional states of the transport cycle. The dxr bound substrate was found in the periplasmic domain of only one of the three protomers. The voluminous binding pocket is aromatic and allows multi-site binding. For binding of 4, the analysis shows 79 distinct conformational clusters of low probability, the largest population being as small as 3. More interestingly, docking of 1 led to 41 conformational clusters, the largest population attaining 16. This strong probability cluster is localized close to the dxr binding site, upstream of the dxr position relative to the entry of the binding pocket (Figure 8c). As this binding pocket has been modeled in Patched, the AcrB-1 and -4 clusters where superposed on the Patched model (Figure 8d). It results that 1 is predicted to interact with Patched in a similar way as dxr binds to AcrB. Then it could be hypothesized that dxr and panicein A hydroquinone could compete by reaching similar binding sites in Patched. This is in good agreement with the inhibition of dxr efflux observed in the presence of panicein A hydroquinone.

## DISCUSSION

Multidrug resistance (MDR) is a phenomenon of resistance of tumors to chemically unrelated anticancer drugs, and is one of the most formidable challenges in the field of cancer chemotherapy [40, 41]. Multidrug resistance can have many causes such as alterations in DNA repair, defective regulation of apoptotic gene expression, enhanced intracellular drug detoxification, but the most common mechanism is the efflux of cytotoxic drugs by membrane transporters. In human, most of the multidrug transporters belong to the large ATP-binding cassette (ABC) transporter super family of membrane proteins from which P-glycoprotein (P-gp) was the first member to be identified [42–44]. Many inhibitors of P-gp have been tested in clinical trials to assess their pharmacological potential. Unfortunately, most of them have failed because they displayed non-specific toxicity [45].

Emerging data from many human tumors have shown that the chemotherapy-resistant phenotype of cancer cells correlates with the activation of Hedgehog (Hh) signaling, and that the Hh pathway regulates cancer stem cells or tumor initiating cells [21, 22, 46, 47]. As an example, Hh signaling is often activated in melanomas and might have a critical role in determining the stem-like characteristics of melanoma initiating cells, contributing to the acquisition of a more undifferentiated and aggressive state through a process similar to reprogramming [48, 49]. The Hh receptor Patched being a Hh target gene, this membrane protein is over-expressed in many recurrent and metastatic tumors such as breast, lung, colorectal, ovarian, prostate cancers or melanoma, as shown by the data extracted from the Human Protein Atlas web site presented in Supplementary Figure 3. We recently discovered that Patched is a multidrug efflux pump that transports different chemotherapeutic agents out of cells, and particularly doxorubicin, using the proton motive force like the bacterial multidrug efflux pumps from the RND family [25]. This is a real breakthrough which suggests that the Hh receptor Patched participates to the resistance to chemotherapy of cancer cells, and allows proposing Patched as a new target for anti-cancer therapy. In contrast to ABC transporters such as P-gp which are ubiquitously expressed, Patched is expressed in adults more specifically in cancer cells. Therefore, inhibitors of Patched drug efflux activity should display less non-specific toxicity than P-gp inhibitors. We then designed innovative screening tests to identify molecules able to inhibit the drug efflux activity of Patched. Compounds extracted from Mediterranean sponges are among the chemical molecules screened to date. Our results show that some natural compounds isolated from the Mediterranean sponge *Haliclona (Soestella) mucosa* inhibit the resistance to the chemotherapeutic agent doxorubicin (dxr) conferred to yeast by the expression of human Patched. Four of these compounds have been identified as meroterpenoids named paniceins, and correspond to panicein A hydroquinone (1), panicein B2 (2), panicein B3 (3) and panicein C (4). We observed that 1 strongly enhanced the dxr cytotoxicity on melanoma cells and increased the number of apoptotic cells in the presence of dxr, while this molecule showed low cytotoxicity by itself. Dxr fluorescence quantification performed on melanoma cells evinced that treatment with 1 increased dxr intracellular concentration. Similar experiments performed on yeast expressing Patched showed that 1 inhibits the dxr efflux activity of Patched. These data are supported by the fact that 1 has no effect on yeast expressing a mutant of Patched which does not transport dxr or on control yeast. The docking realized on the structure of the *E. coli* drug efflux pump AcrB and the structural model of Patched indicated that 1 has a strong probability of interacting close to the dxr binding site, suggesting that this molecule could prevent dxr efflux. Our results suggest that 1 increases the cytotoxic effect of dxr by inhibiting the efflux activity of Patched. The four paniceins identified in our study have already been assayed for cytotoxicity against tumoral cell lines [31]. These molecules were shown to exhibit moderate cytotoxicity above the micromolar range with 1 being the most selective against CCRF-CEM leukemia cells (with a growth inhibitory power (GI_50_) of 7.8 μM, a cytostatic effect (TGI) of 25 μM and a cytotoxic effect (LC_50_) lees than to 25 μM). These previous observations together with our present results underline the therapeutical potential of these compounds. The high reactivity of the hydroquinone could hamper the direct use of paniceins as therapeutic agents. However, other terpene quinones from marine origin, *e.g*. avarol or ilimaquinone [50], have also been shown to exhibit a wide range of interesting bioactivity. In the case of paniceins, this is the first report of a strong therapeutic potential. Moreover, due to the quite easy access to organic synthesis of these molecules [51], slight modifications of the hydroquinone could enhance the bioactivity and interest of panicein analogues as therapeutic agents. In a more general context, these studies underline the importance to assess the biological effects of already known natural substances on a large array of biological targets and the key chemical features of marine natural products [52].

Taken together, our results evidence that panicein A hydroquinone (1) isolated from the Mediterranean sponge *Haliclona (Soestella) mucosa* is the first inhibitor of the dxr efflux activity of Patched. The increase of dxr cytotoxicity induced by panicein A hydroquinone on two melanoma cell lines provides evidence for the relevance of Patched as a therapeutic target in a subset of melanoma, and suggests that the use of inhibitors of Patched drug efflux activity in combination with classical chemotherapy may be a good approach to circumvent tumor drug resistance and to increase cytotoxicity of chemotherapy toward cancer stem cells and the corresponding differentiated tumor cells. This multimodal strategy may represent a breakthrough and the next challenge in cancer treatment.

## MATERIALS AND METHODS

### Chemical procedure

Methanol (MeOH, CHROMASOLV for HPLC), dichloromethane (DCM, CHROMASOLV for HPLC), acetonitrile (ACN, CHROMASOLV for HPLC) and trifluoroacetic acid (TFA) were purchased from Sigma. CD_3_OD for NMR was purchased from Eurisotop. Bulk RP-C18 solid phase was Polygoprep 60–50 purchased from Macherey-Nagel. HPLC purification was performed on a Jasco system equipped with an interface Jasco LC-Net II/ADC, a Jasco UV-2075 Plus detector, two Jasco PU 2087 Plus pumps and an ELSD Sedex 85 detector from SEDERE. NMR spectra were recorded at 25°C on a Bruker Avance 500 spectrometer at 500 MHz (^1^H). Chemical shifts are reported in ppm using residual CD_3_OD (δ3. 31 for ^1^H) as internal reference.

### Biological material

*Cymbaxinella verrucosa, Ircinia variabilis, Ircinia oros, Agelas oroides, Cymbaxinella damicornis, Aplysina cavernicola, Haliclona (Soestella) mucosa, Phorbas topsenti, Chondrosia reniformis, Chondrilla nucula, Haliclona (Halichoclona) fulva, Crambe crambe, Haliclona (Rhizoniera) sarai, Acanthella acuta, Scopalina lophyropoda* sponge individuals (max 10 g wet weight) were collected by hand using SCUBA diving in a cave at Villefranche-sur-Mer (France) at depths ranging from 10 or 20 m, and kept frozen until used.

Human melanoma cell lines MEWO and A375 were purchased from ATCC. The two cell lines were grown in DMEM medium supplemented with 10% FBS, 100 U/ mL penicillin and 100 mg/mL streptomycin, at 37°C in a 5% CO2/95% air water-saturated atmosphere.

K699 *Saccharomyces cerevisiae* yeast strain (Mata, ura3, and leu 2–3, kindly donated by R. Arkowitz) were transformed with the following expression vector: pYEP-hPatched-MAP (giving yeast expressing human Patched), pYEP-mMyo-MAP (for control), or pYEP-hPatchedG509VD513Y-MAP (giving yeast expressing mutant PatchedVXXXY), and grown as described [35] at 18°C until optical density (OD) at 600 nm reached 5 to 7.

### Extraction and purification

For the first screening of sponge extracts, sponge samples (5–10 g wet weight) were freeze dried and then ground to obtain a dry powder which was extracted with a mixture of DCM/MeOH (1:1, v/v) in an ultrasonic bath to obtain an extract after concentration under reduced pressure. This extract was fractionated by Solid Phase Extraction (Strata^®^ C18-E, 2 g, Phenomenex) with a step gradient of H_2_O, MeOH and DCM (14 mL each).

To identify the compounds responsible for the bioactivity, 40.3 g of the freeze-dried sponge *Haliclona (Soestella) mucosa* was extracted with a mixture of DCM/MeOH (1:1, v/v) (400 mL) during 15 min in an ultrasonic bath and the supernatant was collected. This step was repeated twice to finally give 5.6 g of extract after concentration under reduced pressure. The extract was fractionated by RP-C18 vacuum liquid chromatography with solvents of decreasing polarity to obtain 5 fractions: H_2_O (F1), H_2_O/MeOH 1:1 (F2), H_2_O/MeOH 1:3 (F3), MeOH (F4) and DCM (F5) (500 mL each). Fraction F4 was purified by C_18_ preparative HPLC (19 mm × 250 mm × 5 μm, Xselect CSH from Waters) with an isocratic elution with H_2_O/ACN/TFA (30:70:0.1) during 18 min leading to 9 pure compounds P1 to P9 (Supplementary Figure 1). Four of them were identified as panicein A hydroquinone (1, P7, 13.0 mg), panicein B2 (2, P6, 5.50 mg), panicein B3 (3, P4, 41.8 mg), and panicein C (4, P3, 29.3 mg) (Supplementary Figure 2).

Panicein A hydroquinone (1): ^1^H NMR (CD_3_OD) δ6.59 (d, 1H, H-3′), 6.56 (s, 1H, H-4), 6.55 (d, 1H, H-6′), 6.45 (dd, 1H, H-4′), 5.39 (t, 1H, *J* = 7.0 Hz, H-10), 3.74 (s, 3H, H-16), 3.27 (d, 2H, *J* = 7.5 Hz H-11), 2.72 (m, 2H, H-7), 2.29 (s, 3H, H-13), 2.21 (s, 3H, H-14), 2.09 (m, 2H, H-8), 2.09 (s, 3H, H-15), 1.81 (s, 3H, H-12).

Panicein B2 (2): ^1^H NMR (CD_3_OD) δ10.33 (s, 1H, H-15), 6.62 (d, 1H, *J* = 8.5 Hz, H-5′), 6.59 (s, 1H, H-4), 6.56 (dd, 1H, *J* = 8.5, 3.0 Hz, H-4′), 6.48 (d, 1H, *J* = 3.0 Hz, H-2′), 6.37 (d, 1H, *J* = 10.0 Hz, H-11), 5.73 (d, 1H, *J* = 10.0 Hz, H-10), 2.77 (m, 2H, H-7), 2.51 (s, 3H, H-14), 2.28 (s, 3H, H-13), 1.42 (s, 3H, H-12), 1.29 (m, 2H, H-8).

Panicein B3 (3): ^1^H NMR (CD_3_OD) δ10.34 (s, 1H, H-15), 6.60 (s, 1H, H-4), 6.58 (d, 1H, *J* = 8.5 Hz, H-3′), 6.54 (s, 1H, *J* = 3.0 Hz, H-6′), 6.45 (dd, 1H, *J* = 8.5, 3.0 Hz, H-4′), 5.38 (t, 1H, *J* = 7.0 Hz, H-10), 3.26 (d, 2H, *J* = 7.5 Hz, H-11), 2.76 (m, 2H, H-7), 2.54 (s, 3H, H-14), 2.33 (s, 3H, H-13), 2.14 (m, 2H, H-8), 1.82 (s, 3H, H-12).

Panicein C (4): ^1^H-NMR (CD_3_OD) δ10.33 (s, 1H, H-15), 6.59 (d, 1H, *J* = 8.5 Hz, H-3′), 6.54 (d, 1H, *J* = 3.0 Hz, H-6′), 6.45 (dd, 1H, *J* = 8.5, 3.0 Hz, H-4′), 5.39 (t, 1H, *J* = 7.0 Hz H-10), 3.27 (d, 2H, *J* = 7.5 Hz, H-11), 2.76 (m, 2H, H-7), 2.48 (s, 3H, H-14), 2.27 (s, 3H, H-13), 2.13 (m, 2H, H-8), 1.82 (s, 3H, H-12).

### Effect of sponge methanolic and purified fractions on the resistance of yeast expressing Patched to doxorubicin

Yeast expressing Patched were grown in 10 mL of minimal medium (supplemented with 2% of glucose and aminoacids cocktail without leucine) at 30°C. Yeast were pre-cultured at 30°C in the same medium to OD_600_ = 1–2, and diluted in rich medium containing 2% of glucose in 96-well plates. Methanolic or purified fractions were dissolved in DMSO at 10 mg/mL and added in all wells at 10 μg/mL (1/1000 dilution) or other final concentrations (for IC50 measurements), doxorubicin (dxr) (10 μM final concentration) was added in half of the wells. DMSO at a dilution of 1/1000 corresponding to the amount of DMSO added with sponge extracts or compounds was added in the control wells. We checked that this amount of DMSO had no effect on yeast growth in the absence or in the presence of dxr (not shown). Plates were incubated at 18°C on a shaker at 1250 rpm (microtitre plate shaker SSL5 Stuart) and absorbance at 600 nm was recorded for about 72 h. IC_50_ were calculated using online fit of log-dose vs response from Graph Pad Prism software.

### Effect of sponges purified fractions on doxorubicin cytotoxicity on melanoma cells

MEWO and A375 melanoma cells were seeded on 96-well plates and grown 48 h in complete DMEM medium to achieve 60% to 70% confluence. Medium was then removed and replaced with 100 μL/well of complete DMEM medium containing the compounds of interest at defined concentration or DMSO as control. After 2 h, 100 μL of complete DMEM medium containing dxr was added in half of the wells to obtain 2 or 1.5 μM dxr. 100 μL of complete DMEM medium without dxr was added in the other half of the plate. Plates were incubated at 37°C in a 5% CO_2_/95% air water-saturated atmosphere. After 24 h, plates were incubated 3 h at 37°C with 100 μL/wells of neutral red (NR) solution (50 μg/mL in DMEM). After a rapid wash with cold PBS, plates were gently tapped several times on absorbent paper. Cells were solubilized with 100 μL of a solution containing 1% acetic acid, 49% H_2_O, 50% ethanol by vortexing 3 min at 700 rpm and the absorbance at 600 nm was measured. IC_50_ were calculated from the mean of at least three experiments for each cell line using online fit of log-dose vs response from Graph Pad Prism software.

### Protein quantification

Protein concentrations were determined by the Bradford method using a Bio-Rad kit.

### SDS-PAGE and western-blotting

Total RIPA extracts from melanoma cells were prepared. Samples were separated on 8% SDS-PAGE and transferred to nitrocellulose membranes (Amersham) using standard techniques. After 1 hr at room temperature (RT) in blocking buffer (20 mmol/L Tris-HCl pH 7.5, 45 mmol/L NaCl, 0.1%Tween-20, and 4% non-fat milk), nitrocellulose membranes were incubated overnight at 4°C with rabbit anti-Patched antiserum (Ab39266 from Abcam 1/1000) and monoclonal mouse anti-βtubulin antibody (Sigma; 1/1000). After 3 washes, membranes were incubated 45 min with anti-mouse (1:5000) or anti-rabbit (1:3000) immunoglobulin coupled to horseradish peroxidase (Dako). Detection was carried out with an ECL kit (Millipore) on a Las3000 (Fuji).

### Immuno-labelling

Cells were seeded on cover slips in 12-well plates and allowed to grow to 80% confluence. Cells were washed twice with PBS and fixed 10 min with 4% paraformaldehyde (PFA, Sigma) and then incubated in PBS supplemented with 0.1% Tween and 1% BSA for 1 h to permeabilize the cells and block non-specific protein-protein interaction. Cells were then incubated with anti-Patched antibody (Ab39266 from Abcam 1/200) overnight at 4°C. Cells were washed with PBS/0.1%BSA before incubation with the secondary antibody (Alexa Fluor 494 goat anti-rabbit IgG, (red)) at 1/200 dilution for 1 h at RT. DAPI was used to stain the cell nuclei (blue). Cells were observed by epifluorescence microscopy (Axioplan2 imaging from Zeiss coupled to a cool SNAP HQ from Roper Scientific) using an objective Plan NeoFluar 40x /1.3.

### Drug efflux measurements

For dxr incorporation in melanoma cells, the protocol was adapted from Bidet *et al*. [25]. Cells were seeded on cover slips in 12-well plates and allowed to grow to 80% confluence. Cover slips were incubated 2 h at 37°C and 5% CO_2_ with 10 μM of dxr in physiological buffer (140 mM NaCl, 5 mM KCl, 1 mM CaCl2, 1 mM MgSO4, 5 mM glucose, 20 mM HEPES, pH 7.4) protected from light and quickly rinsed with phosphate buffer (pH 7.4). One cover slip of each cell line was immediately fixed 10 min with 4% PFA for dxr loading control. The other cover slips were incubated with physiological buffer supplemented with DMSO or 10 μM of paniceins 30 min under gentle shaking at 37°C and 5% CO_2_ protected from light, and immediately fixed with 4% PFA. Cells were observed by epi-fluorescence microscopy (λex: 485 nm, λem: 600 nm) (Axioplan2 imaging from Zeiss coupled to a cool SNAP HQ from Roper Scientific) using an objective Plan NeoFluar 40x /1.3.

For dxr incorporation in yeast, yeast expressing Patched, mutant Patched (PatchedVXXXY), or control yeast were washed with cold water, resuspended at an OD_600_ of 10 in Hepes–NaOH buffer (pH 7.0) supplemented with 5 mM of 2-deoxy-D-glucose to inhibit glycolysis and de-energize yeast, and incubated with 10 μM dxr for 2 h at 4°C in the cold room on a rotating wheel in the dark. Yeasts were centrifuged and the supernatant was removed. One sample was immediately fixed with 4% PFA for dxr loading control. The other samples were resuspended in Hepes–NaOH buffer (pH 7.0) containing 5 mM of 2-deoxy-D-glucose supplemented with DMSO or 10 μM of paniceins, and incubated 10 min at 25°C with gentle shaking in a Benchmark Multi-therm shake protected from light. Samples were centrifuged for 1 min at 18, 000 g, supernatants were removed and yeast were fixed with 4% PFA. 10 μL of each sample were deposited on a cover slip and observed by epi-fluorescence microscopy (λex: 485 nm, λem: 600 nm) using an objective Plan NeoFluar 63x /0.7–1.25 Iris.

Quantification of dxr intracellular fluorescence was carried out using Image J software on more than 30 cells or yeasts from 3 different fields for each condition. The results were analyzed using the Student *t*-test in which significance is attained at *P* < 0.05.

### Apoptosis measurement

Apoptosis measurements were carried out using Annexin V conjugated to allophycocyanin (APC) (BD Pharmingen) and DAPI labeling. Cells were cultivated in 6 well-plates to 50% confluence, and treated or not with paniceins and/or dxr for 24 h at 37°C and 5% CO_2_. Cells were washed twice with cold PBS and dissociated by incubation with PBS containing 2% EDTA 5 to 10 min at 37°C and 5% CO_2_. After centrifugation, cells were resuspended with 100 μl of Annexin V binding buffer (BD Pharmingen) and incubated with 2.5 μL of APC Annexin V and/or 3 μL of DAPI (5 mg/mL) 15 min at 25°C in the dark. 100 to 200 μl of binding buffer was added to each tube and flow cytometry analysis was carried out within 1 h using a LSR II Fortessa de BD (BD Biosciences). The untreated population was used to define the basal level of apoptotic and dead cells. The percentage of cells that have been induced to undergo apoptosis was then determined by subtracting the percentage of apoptotic cells in the untreated population from percentage of apoptotic cells in the treated population. Cells in the late stages of apoptosis have a damaged membrane and stain positive for DAPI as well as for APC Annexin V.

### Patched 3D model

Three-dimensional models of Patched were designed by using three different programs: the web modeling pipeline @TOME-2 (http://atome.cbs.cnrs.fr/AT2B/meta.html [53], Phyre2 (http://www.sbg.bio.ic.ac.uk/phyre2/html/page.cgi?id=index [54] and I-TASSER (http://zhanglab.ccmb.med.umich.edu/I-TASSER/) [55, 56], the winner of the last CASP competitions (Critical Assesment for Structural Prediction) [57]. For the @TOME-2 model building, the first step consists in a fold-recognition search and issues with a sequence alignment with proteins whose structures have been solved and deposited in the Protein Data Bank (PDB: http://www.rcsb.org/pdb/home/home.do). A careful analysis of this sequence alignment is then performed, leading to the removing of several protein structures from the data to be used for the second step. The second module of @TOME then run MODELLER (https://salilab.org/modeller/) [58] for model building. Concerning Phyre2 and I-TASSER, the two programs were used without manual intervention nor geometry refinement.

These different trials for Patched model building revealed mainly membrane protein transporters from the RND family (AcrB, MexB, CusA) but some other unrelated protein structures were also suggested that differed between the three programs results (like for example an helicase (PDB code 4F91; [59]), or the translocon-associated membrane protein SecDF (PDB code 3AQP; [60]). Even if the three different programs were mainly used with the automatic procedures, each possible resulting model was analyzed, and all those with low sequence coverage and/or percentage of identity with the target used lower than 10% were omitted. In addition it was verified that the location of the transmembrane segments was compatible with the prediction of TM performed by HMMTOP (http://www.enzim.hu/hmmtop/), TOPPRED (http://mobyle.pasteur.fr/cgi-bin/portal.py?#forms::toppred) and TMHMM (http://www.cbs.dtu.dk/services/TMHMM/), and that the GXXXD motif corresponding to the proton transfer sequence was localized in a transmembrane segment. It has also been verified that the sequence duplication of human Patched corresponds to structure duplication in the model.

The use of several programs for cross validation was motivated by the low sequence identities between human Patched and the proteins used in the model building, 17% with CusA, 18% with AcrB (36% of sequence similarity), 18.4% with MexB, 19% with Czca, 16% with Mtrd, all pertaining to the RND proteins family. Even if those values are not high, it has to be noticed that for example the sequence identity between AcrB and CusA that share the same structure (RMSD = 4.2Å on 823 aligned Cα atoms), is only 21%. Even if differences exist between the models issued from the different programs, especially in the extracellular domain, they all model the same portion of Patched sequence, from 94 to 1183. The transmembrane region is the most conserved one; in particular the sequence ^509^GVGVD^513^, corresponding to the proton transfer pathway, is perfectly aligned in all the models (Figure 8a). A separate analysis of the N- and C-terminal domains of Patched was performed with PONDR [61] predicting those two domains as being mainly disordered. The final Patched model, corresponding to the second model proposed by I-Tasser, results mainly from the structure of AcrB as evidenced from structural superposition (Figure 8b).

### Docking

The docking of two panicein molecules (1 and 4) on the 2.9 Å resolution AcrB structure (PDB code 2GIF; [38]) was performed using AutoDock [62]. For each molecule seven active torsions were chosen. Several runs were performed using a grid search of 126 points in each direction with a grid spacing of 0.44 Å, localized on one of the three monomers forming the functional trimeric AcrB unit, the one in the loose state permitting the entry of molecules in the groove. For each calculation, 100 genetic algorithm runs were performed with a population size of 150. The docking of 4 on AcrB led to 79 distinct conformational clusters, the largest population being as small as 3. For docking of 1, 41 conformational clusters were found out of the 100 runs, the largest population attaining 16. This cluster of 16 ranks at the 4th lowest binding energy level, but close to the first one that is only composed of 3 different poses.

## SUPPLEMENTARY FIGURES


