# Supplementary material for: Natural paniceins from mediterranean sponge inhibit the multidrug resistance activity of Patched and increase chemotherapy efficiency on melanoma cells
Source: Oncotarget. 2015 Jun 1;6(26):22282–97. doi: 10.18632/oncotarget.4162 (PMC4673163; doi:10.18632/oncotarget.4162)
Supplement: Supplementary file 1 [file oncotarget-06-22282-s001.pdf]

SUPPLEMENTARY FIGURES

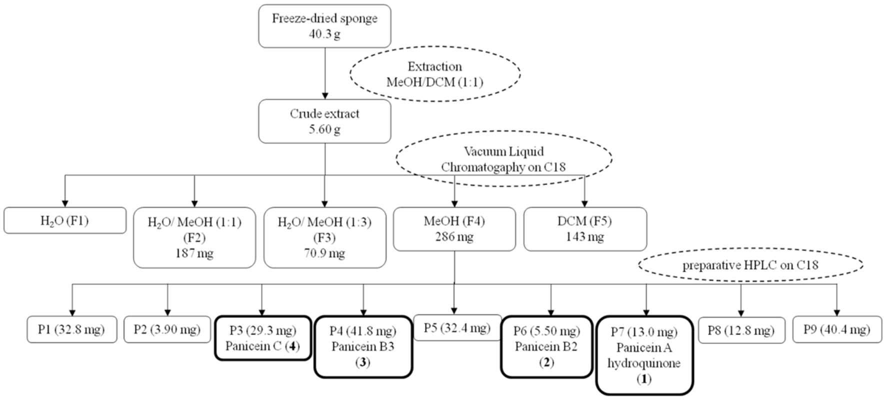

Supplementary Figure S1: Purification scheme of the four paniceins isolated from the Mediterranean sponge *Haliclona mucosa.*

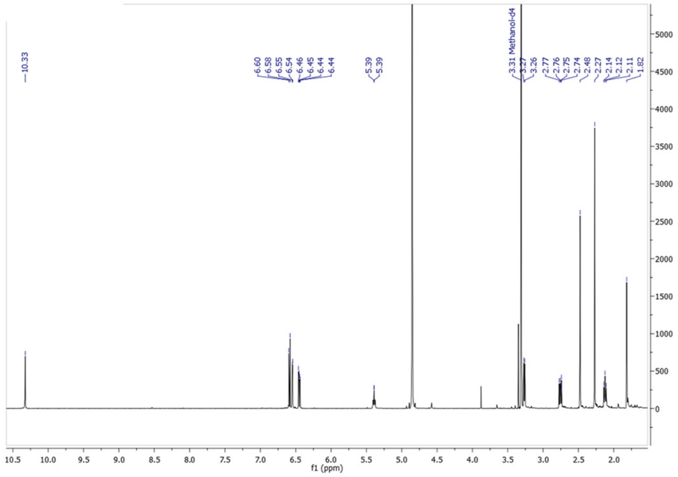

Supplementary Figure S2a: NMR spectrum of panicein C (4, F4P3) in CD_3OD at 500 MHz.

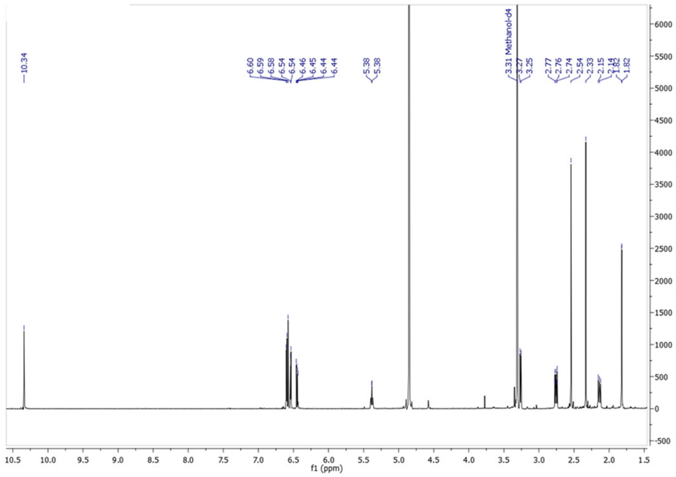

Supplementary Figure S2b: NMR spectrum of panicein C (3, F4P4) in CD_3OD at 500 MHz.

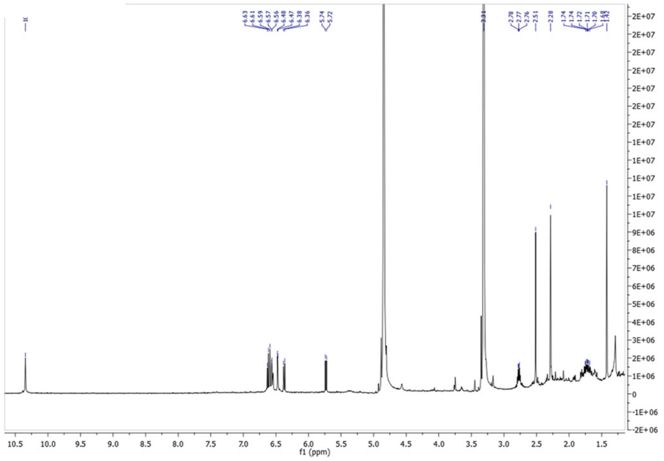

Supplementary Figure S2c: NMR spectrum of panicein B2 (2, F4P6) in CD_3OD at 500 MHz.

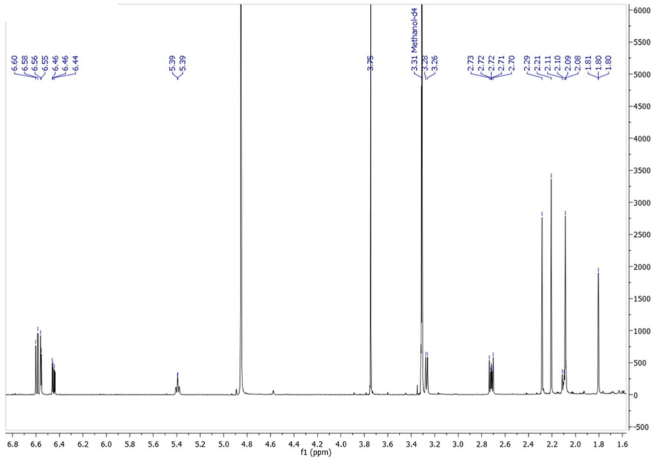

Supplementary Figure S2d: NMR spectrum of panicein A hydroquinone (1, F4P7) in CD_3OD at 500 MHz.

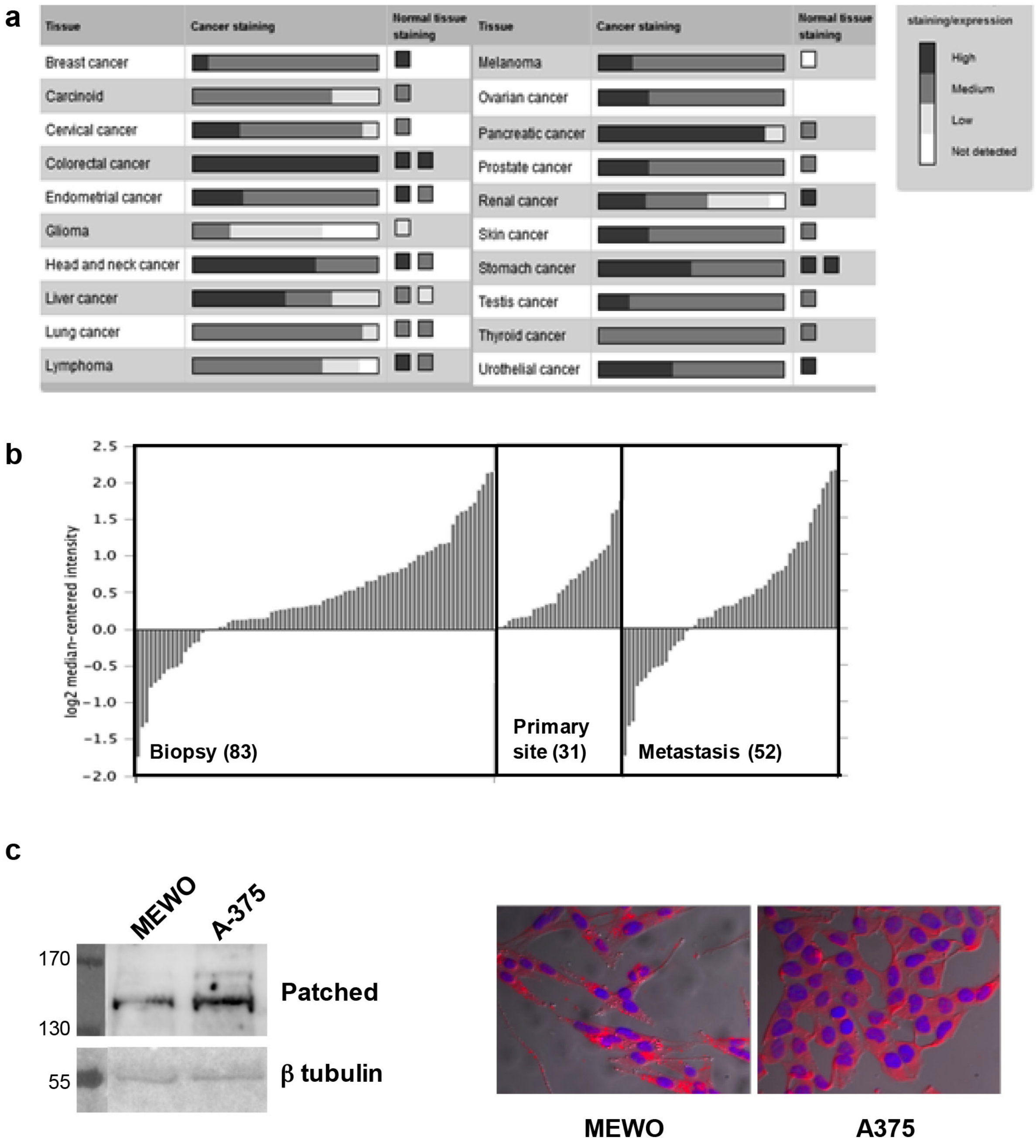

Supplementary Figure S3: Expression of Patched in Melanoma. a. Patched protein is expressed in different cancers (IHC on tissue, data extracted from the Human Protein Atlas web site). **b.** Patched mRNAs in 154 melanoma samples (data extracted from ONCOMINE web site). **c.** Patched protein is expressed in MEWO and A375 melanoma cell lines. Left: western-blotting on total extracts from MEWO and A375. Right: Patched immuno-labeling (in red) of MEWO and A375 cells was superposed to DAPI (in blue) and contrast phase image of the cells. Rhodamine-anti-rabbit antibodies alone did not give any signal (not shown).

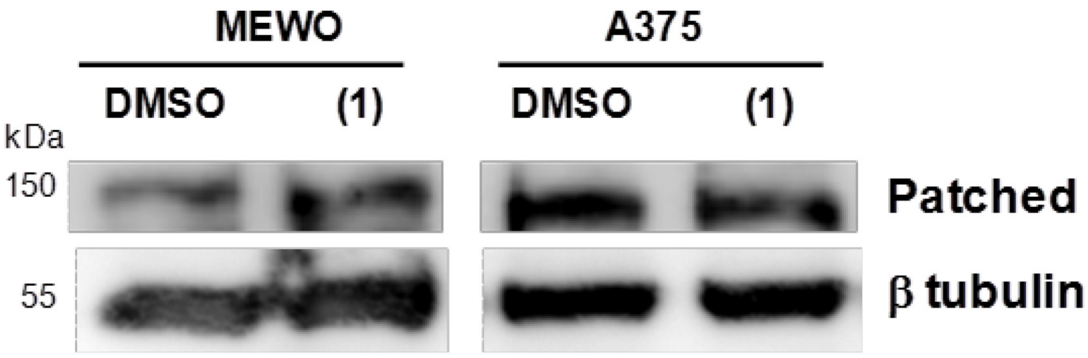

Supplementary Figure S4: Panicein A hydroquinone does not affect Patched protein expression or stability. Western-blotting on total extracts from MEWO and A375 cells after 24 h treatment with 10 µM of panicein A hydroquinone or with DMSO.
